# p53 directly downregulates the expression of CDC20 to exert anti-tumor activity in mantle cell lymphoma

**DOI:** 10.1186/s40164-023-00381-7

**Published:** 2023-03-07

**Authors:** Yingtong Chen, Ping Yang, Jing Wang, Shuang Gao, Shiyu Xiao, Weilong Zhang, Mingxia Zhu, Yanfang Wang, Xiaoyan Ke, Hongmei Jing

**Affiliations:** 1 Department of Hematology, Lymphoma Research Center, Peking University Third Hospital, 49 Huayuan North Road, Haidian District, Beijing, 100191 China; 2 Department of Gastroenterology, Peking University Third Hospital, 49 Huayuan North Road, Haidian District, Beijing, 100191 China; 3 Center of Basic Medical Research, Institute of Medical Innovation and Research, Peking University Third Hospital, 49 Huayuan North Road, Haidian District, Beijing, 100191 China

**Keywords:** Mantle cell lymphoma, p53, CDC20, Proliferation, Apoptosis

## Abstract

**Background:**

Cell cycle dysregulation characterized by cyclin D1 overexpression is common in mantle cell lymphoma (MCL), while mitotic disorder was less studied. Cell division cycle 20 homologue (CDC20), an essential mitotic regulator, was highly expressed in various tumors. Another common abnormality in MCL is p53 inactivation. Little was known about the role of CDC20 in MCL tumorigenesis and the regulatory relationship between p53 and CDC20 in MCL.

**Methods:**

CDC20 expression was detected in MCL patients and MCL cell lines harboring mutant p53 (Jeko and Mino cells) and wild-type p53 (Z138 and JVM2 cells). Z138 and JVM2 cells were treated with CDC20 inhibitor apcin, p53 agonist nutlin-3a, or in combination, and then cell proliferation, cell apoptosis, cell cycle, cell migration and invasion were determined by CCK-8, flow cytometry and Transwell assays. The regulatory mechanism between p53 and CDC20 was revealed by dual-luciferase reporter gene assay and CUT&Tag technology. The anti-tumor effect, safety and tolerability of nutlin-3a and apcin were investigated in vivo in the Z138-driven xenograft tumor model.

**Results:**

CDC20 was overexpressed in MCL patients and cell lines compared with their respective controls. The typical immunohistochemical marker of MCL patients, cyclin D1, was positively correlated with CDC20 expression. CDC20 high expression indicated unfavorable clinicopathological features and poor prognosis in MCL patients. In Z138 and JVM2 cells, either apcin or nutlin-3a treatment could inhibit cell proliferation, migration and invasion, and induce cell apoptosis and cell cycle arrest. GEO analysis, RT-qPCR and WB results showed that p53 expression was negatively correlated with CDC20 expression in MCL patients, Z138 and JVM2 cells, while this relationship was not observed in p53-mutant cells. Dual-luciferase reporter gene assay and CUT&Tag assay revealed mechanistically that CDC20 was transcriptionally repressed by p53 through directly binding p53 to CDC20 promoter from − 492 to + 101 bp. Moreover, combined treatment of nutlin-3a and apcin showed better anti-tumor effect than single treatment in Z138 and JVM2 cells. Administration of nutlin-3a/apcin alone or in combination confirmed their efficacy and safety in tumor-bearing mice.

**Conclusions:**

Our study validates the essential role of p53 and CDC20 in MCL tumorigenesis, and provides a new insight for MCL therapeutics through dual-targeting p53 and CDC20.

## Introduction

Mantle cell lymphoma (MCL) is a rare, aggressive and heterogeneous B-cell non-Hodgkin’s lymphoma, with the incidence accounting for about 6% of all non-Hodgkin’s lymphoma and a median age of 68 years old at initial diagnosis [1, 2]. The clinical presentations of MCL are diverse and there are no curative methods up to date, as most MCL patients relapse shortly after initial standard chemotherapy [3]. Although targeted drugs such as Bruton’s tyrosine kinase (BTK) inhibitor [4, 5], phosphoinositide 3-kinase (PI3K) inhibitors [6, 7], B cell lymphoma-2 (BCL2) inhibitors [8–10] and Poly ADP-ribose polymerase (PARP) inhibitors [11] are developed for the treatment of relapsed/refractory MCL, the prognosis of MCL patients still remains poor. Further studies on MCL pathogenesis are conducive to discovering novel small-molecular agents for improving the clinical outcomes of MCL patients.

Cell cycle dysregulation is common in MCL, manifested by the accumulation of aberrant cyclin D1 caused by chromosomal translocation t(11;14)(q13;q32), thus accelerating the transition from G1 phase to S phase and promoting malignant B cell proliferation [12, 13]. However, limited studies focused on mitotic disorder in MCL. Effective anti-tumor strategies that targeting mitosis were reported, which led to mitotic cell arrest and induced mitotic cell death [14]. Cell division cycle 20 homologue (CDC20), an essential mitotic regulator, could bind to the anaphase-promoting complex/cyclosome (APC/C) and activate APC/C to ensure chromosome segregation and facilitate metaphase-anaphase transition, followed by mitotic exit [15]. Plenty of studies have proved that CDC20 functioned as the carcinogenic factor and was highly expressed in a variety of solid tumors and hematological malignancies, including breast cancer [16], lung cancer [17], gastric cancer [18], hepatocellular carcinoma [19], colorectal cancer [20], pancreatic cancer [21], bladder cancer [22], oral squamous cell carcinoma [23], ovarian cancer [24], glioblastoma [25], multiple myeloma [26] and diffuse large B-cell lymphoma [27]. CDC20 overexpression was also closely related to poor pathological classification and shorter survival in these tumor patients, suggesting that it might serve as an important prognostic factor and a potential anti-tumor target. Currently, few studies have explored the role of CDC20 in the development and progression of MCL.

In addition to cell cycle dysregulation, another common abnormality in MCL is p53 inactivation caused by p53 deletion or mutation. As a tumor suppressor, p53 is activated when undergoing endogenous and exogenous cellular stress, and can prevent normal cells from becoming cancerous by triggering cell cycle arrest, apoptosis, cellular senescence or autophagy [28, 29]. Approximately 30% of MCL patients suffered from p53 inactivation, and these patients usually had adverse clinical outcomes [12]. Since p53 inactivation was mediated by MDM2 overexpression in some MCL patients, one way to reactivate p53 was to inhibit MDM2, which was a negative regulator of p53 [30]. Besides, targeted genes that transcriptionally repressed by p53 were frequently overexpressed in tumors [31], and Zhang et al. found p53 increased accompanied with CDC20 decreased after drug treatment in triple-negative breast cancer [32], we hypothesized that a negative regulatory relationship between p53 and CDC20 also existed in MCL.

In this study, we aimed to explore the role of p53 and CDC20 in MCL, demonstrate the specific mechanism on how p53 regulated CDC20, and verify the efficacy and safety of anti-MCL therapy by targeting p53 and CDC20 in vitro and in vivo. The relationship between CDC20 expression level and the clinicopathological features and prognosis of MCL patients was investigated. CDC20 inhibitor apcin and p53 agonist nutlin-3a were used to confirm the effect of inhibiting CDC20 or activating p53 on cell proliferation, apoptosis, cell cycle, migration and invasion abilities of MCL cells. Dual-luciferase reporter gene assay and CUT&Tag technology were performed to clarify the regulatory mechanism between p53 and CDC20. Most importantly, we attempted to prove the combined anti-tumor effect of nutlin-3a and apcin in MCL cells and MCL xenograft model, so as to provide the initial evidence for the clinical feasibility of dual-targeting p53 and CDC20 in MCL therapeutics.

## Materials and methods

### Acquisition of clinical samples

Peripheral blood from 24 MCL patients and 7 healthy controls as well as bone marrow from 17 MCL patients and 10 healthy donors were collected at Peking University Third Hospital from December 2020 to September 2021. Peripheral blood mononuclear cells (PBMCs) and bone marrow mononuclear cells (BMNCs) were extracted from peripheral blood and bone marrow using Ficoll Plus 1.077 solution (Solarbio, Beijing, China), respectively. Sections of 51 MCL patients with tumor tissues were obtained from Department of Pathology of Peking University Third Hospital between January 2010 to June 2021, and another 12 sections with lymph node reactive hyperplasia (LRH) were served as their controls. The clinical data of the above patients were recorded for analysis. This study was approved by the Ethics Committee of Peking University Third Hospital (S2021251), and all the participants signed informed consent.

### Cell lines and cell culture conditions

Four human MCL cell lines Jeko, Mino, Z138 and JVM2 were used in this study, which were all purchased from the American Type Culture Collection (ATCC, USA) and performed short tandem repeat (STR) identification. Among these cell lines, Z138 and JVM2 expressed wild-type p53, whereas Jeko and Mino carried mutant p53 [Jeko: p.Pro58Glnfs*65 (c.173delC); Mino: p.Val147Gly (c.440 T > G). The mutation information was obtained from Cellosaurus database (https://www.cellosaurus.org/)] cell were cultured in RPMI-1640 medium (Cytiva, USA) with 10% (Mino, Z138 and JVM2) or 20% (Jeko) fetal bovine serum (FBS, Gibco, USA) and 1% penicillin–streptomycin (Gibco, USA) in a 37℃ humidified incubator containing 5% CO_2_.

### Cell viability assay

Cell viability was determined by Cell Counting Kit-8 assay (CCK-8, APExBIO, USA). Cells were inoculated in 96-well plates at a density of 10^4^ cells per well in 100 μl complete medium (RPMI-1640 medium + 10%FBS + 1% penicillin–streptomycin). Cells were exposed to designated concentrations of CDC20 inhibitor apcin (Selleck, USA) or p53 agonist nutlin-3a (Selleck, USA) alone, or co-treated with apcin and nutlin-3a, and incubated for 24 h, 48 h and 72 h. Nutlin-3a could inhibit the interaction between MDM2 and p53 and activate p53^33^, so we identified nutlin-3a as the p53 agonist in this study. At each time point, 10 μl of CCK-8 solution was added to each well, and then incubated for another 4 h. After that, the optical density (OD) value per well was measured at 450 nm using a microplate reader (Tecan Spark, Switzerland). Cell viability at each time point was defined as the ratio of the OD value in the agent-treatment group to that in the corresponding untreated group. In contrast, inhibitory rate = 1-cell viability. Whether combination of apcin and nutlin-3a had synergistic effect was determined by combination index (CI) values calculated by CompuSyn software (ComboSyn, Inc., USA) based on the inhibitory rate of the two agents.

### EdU cell proliferation assay

Cell proliferation was assessed using the BeyoClick™ EdU Cell Proliferation Kit (Beyotime, Shanghai, China). Cells were inoculated in 24-well plates at a density of 5 × 10^5^ cells per well in 1 ml complete medium, and then were treated with different concentrations of apcin or nutlin-3a alone, or co-treated with apcin and nutlin-3a. After cultured for 48 h, 10 μM EdU reagent was added to each well, and the plates were incubated for another 2 h. The stained cells were detected by flow cytometry (Beckman, USA). CytExpert software (Beckman, USA) was used to analyze the EdU incorporation rate, which reflected cell proliferation ability.

### Cell apoptosis analysis

Cell apoptosis was examined using Annexin V-FITC/PI Apoptosis Detection Kit (Vazyme, Nanjing, China). Cells were inoculated in 24-well plates at a density of 4 × 10^5^ cells per well in 1 ml complete medium. These cells were treated with different concentrations of apcin or nutlin-3a alone, or co-treated with apcin and nutlin-3a. Cells were collected by centrifugation after 48 h, and incubated with 5 μl Annexin V-FITC and 5 μl PI at the room temperature for 10 min in the dark. The stained cells should be detected by flow cytometry (BD, USA) within 1 h. FlowJo software (Version10.6.2, FlowJo LLC, BD, USA) was used to analyze the total apoptosis rate, which was the sum of the early apoptosis rate and the late apoptosis rate.

### Mitochondrial membrane potential detection

The Mitochondrial Membrane Potential Assay Kit with JC-1 (Solarbio, Beijing, China) was used to detect the changes of mitochondrial membrane potential (MMP) in cells, as well as evaluate the early cell apoptosis. When MMP is high, JC-1 probe appears as aggregates and emits red fluorescence; when MMP is low, JC-1 probe appears as monomers and emits green fluorescence. The transition from red to green fluorescence represents the decrease in MMP, which is a hallmark event of early apoptosis. Cells were inoculated in 24-well plates at a density of 4 × 10^5^ cells per well in 1 ml complete medium. These cells were treated with different concentrations of apcin or nutlin-3a alone, or co-treated with apcin and nutlin-3a. Cells were harvested after 48 h, resuspended in 0.5 ml complete medium mixed with 0.5 ml JC-1 staining solution, and incubated at 37 ℃ for 20 min. The stained cells were detected by flow cytometry (Beckman, USA). The results were displayed as the ratio of mean red fluorescence intensity to mean green fluorescence intensity analyzed by CytExpert software (Beckman, USA).

### Cell cycle analysis

Cell cycle was evaluated using the Cell Cycle Detection Kit (KeyGEN, Nanjing, China). Cells were inoculated in 24-well plates at a density of 4 × 10^5^ cells per well in 1 ml complete medium. Cells were treated with different concentrations of apcin or nutlin-3a alone, or co-treated with apcin and nutlin-3a. Cells were collected after 48 h or 72 h, and resuspended in pre-cooling 70% ethanol and fixed overnight at 4 ℃. On the next day, cells were harvested and resuspended in 500ul staining solution mixed with RNase A and PI (RNase A:PI = 1:9), and incubated at 37 ℃ for 30 min in the dark. The stained cells were detected by flow cytometry (BD, USA). Modfit LT software (Version5.0, Verity Software House, USA) was used to analyze the proportion of G0/G1, S and G2/M phases in the cell cycle.

### Transwell cell migration and invasion assays

Transwell chambers with 24 wells and the pore size of 8 μm (Corning, USA) were used to test cell migration and invasion abilities. A total of 10^5^ cells for the migration experiment or 5 × 10^5^ cells for the invasion experiment was inoculated in the upper chamber in 200 μl serum-free RPMI-1640 medium, while 600ul complete medium was added to the lower chamber. The upper and lower chamber of one well contained the same concentration of apcin or nutlin-3a. After incubation at 37 ℃ for 48 h, the migratory cells or invasive cells that fell from the upper chamber into the lower chamber were counted. Eight images were randomly captured for each group at 10 × magnification under an inverted microscope (Leica, Germany), and cells on each image were counted assisted with Image J software (Version 1.4.3.67). The number of migratory cells or invasive cells of each group was the average cell number on these eight images. For the invasion experiment, 50 μl diluent Matrigel (Corning, USA) in RPMI-1640 medium was pre-coated in the upper chamber before cell seeding, and then incubated at 37 ℃ for 30 min to form the membrane.

### RNA extraction and real-time quantitative polymerase chain reaction (RT-qPCR)

Total RNA was extracted from various cell samples by Trizol reagent (Ambion, Thermo Fisher Scientific, USA) according to the manufacturer's instructions. The concentration and purity of each RNA sample were measured by NanoDrop ONEC (Thermo Fisher Scientific, USA). Then, 1 μg total RNA per cell sample was reverse transcribed into cDNA using Hifair^®^ III 1st Strand cDNA Synthesis SuperMix (Yeasen, Shanghai, China) on a nucleic acid amplifier (Applied Biosystems, Thermo Fisher Scientific, USA). PCR amplification procedure was performed on a RT-qPCR instrument (Quantstudio 5, Applied Biosystems, Thermo Fisher Scientific, USA) using Hieff® qPCR SYBR Green Master Mix (Yeasen, Shanghai, China). The relative expression of mRNA was calculated by the 2^−ΔΔCt^ method, with β-actin as the reference gene. All primers were synthesized by Ruibiotech Company (Beijing, China) and their sequences [27, 34, 35] were listed in Table 1.

**Table 1:** Primer Sequences

Primer name	Sequence
CDC20^34^	Forward 5ʹ-CTGGATCAAAGAGGGCAACTA-3ʹ
	Reverse 5ʹ-GGCAGAGTGACTGGTCATATT-3ʹ
TP53^27^	Forward 5ʹ-ACCGGCGCACAGAGGAAGAG-3ʹ
	Reverse 5ʹ-GCCTCATTCAGCTCTCGGAACATC-3ʹ
β-actin^35^	Forward 5ʹ-GCGTGACATTAAGGAGAAG-3ʹ
	Reverse 5ʹ-GAAGGAAGGCTGGAAGAG-3ʹ

### Protein extraction and western blot (WB) analysis

Total protein was extracted from cell samples using RIPA lysis buffer (APPLYGEN, Beijing, China) mixed with the protease inhibitor (APExBIO, USA) and the phosphatase inhibitor (APExBIO, USA). Protein concentration was quantified by the BCA Protein Assay Kit (Beyotime, Shanghai, China). The equal amount of loaded protein from each sample was separated by 10% or 12.5% SDS-PAGE gels (Biotides, Beijing, China) under electrophoretic conditions and transferred to the polyvinylidene difluoride (PVDF, Merck Millipore, USA) membranes pre-activated with methanol. The PVDF membranes were then blocked with 5% non-fat milk (APPLYGEN, Beijing, China) for 1 h to avoid non-specific binding and incubated with primary antibodies overnight at 4℃. The primary antibodies used were as follows: anti-p53 (1:1000, sc-126, Santa Cruz, USA), anti-CDC20 (1:1000, sc-13162, Santa Cruz, USA), anti-p21 (1:2000, #2947, CST, USA), anti-caspase-3 (1:1000, #14220, CST, USA), anti-cleaved caspase-3 (1:1000, #9664, CST, USA), anti-caspase-9 (1:1000, #9502, CST, USA), anti-cleaved caspase-9 (1:1000, A5074, Bimake, USA), anti-PARP (1:1000, #9532, CST, USA), anti-cleaved PARP (1:1000, #5625, CST, USA), anti-Noxa (1:1000, BM5042, Boster, China), anti-Puma (1:1000, bs-1573R, Bioss, China), anti-MMP2 (1:1000, 10373-2-AP, Proteintech, China), anti-MMP9 (1:1000, 10375-2-AP, Proteintech, China), anti-β-actin (1:15000, 66009-1-Ig, Proteintech, China). On the next day, the membranes were washed with 1 × TBST (APPLYGEN, Beijing, China), and then incubated with horseradish peroxidase-conjugated secondary antibodies (goat anti-mouse IgG or goat anti-rabbit IgG, 1:10000, Zhongshan Goldbridge Biotechnology, Beijing, China) for 1 h at room temperature. Super ECL Detection Reagent (Yeasen, Shanghai, China) was used to detect the protein bands on a chemiluminescence apparatus (Tanon-5200Multi, Shanghai, China). The relative protein expression of each protein band was analyzed by Image J software (Version 1.4.3.67), using β-actin as the reference.

### Dual-luciferase reporter gene assay

A dual-luciferase reporter gene assay system was applied to confirm whether p53 could regulate the activity of CDC20 promoter. TP53 (1182 bp) overexpression plasmid (pcDNA3.1-TP53) was constructed. CDC20 promoter sequence (TSS-1495 bp ~  + 26 bp, TSS: transcription start site) was cloned into pGL4-basic vector as a promoter luciferase plasmid (pGL4-CDC20). Empty pcDNA3.1 and pGL4 vectors were served as the control plasmids. 293 T cells were inoculated into 24-well plates at a density of 2 × 10^5^ per well, and these plasmids were transfected into 293 T cells with the transfection reagent Lipofectamin 2000 (Invitrogen, Thermo Fisher Scientific, USA). The groups were divided as follows: (1) pcDNA3.1 + pGL4 + RL-TK; (2) pcDNA3.1 + pGL4-CDC20 + RL-TK; (3) pcDNA3.1-TP53 + PGL4 + RL-TK; (4) pcDNA3.1-TP53 + pGL4-CDC20 + RL-TK. Among them, *renilla* luciferase reporter gene (RL-TK) was used as the control reporter gene. After 24 h incubation at 37℃, the Promega Dual-Luciferase Reporter Assay System (Promega, USA) was utilized to detect the firefly luciferase activitity and *renilla* luciferase activitity. The relative luciferase activity was expressed as the ratio of firefly luciferase activity to *renilla* luciferase activity.

### CUT&Tag analysis

Cleavage Under Targets and Tagmentation (CUT&Tag) is a new technique for investgating the interaction between proteins and DNA fragments. In this study, CUT&Tag technique was used to explore whether CDC20 was a downstream target gene regulated by p53, and the binding site of p53 on CDC20 promoter in Z138 cells treated with or without 5 μM nutlin-3a. Cells were harvested and the cell nuclei were extracted and purified, followed by incubating p53 primary antibody (1:200, #2524, CST, USA) overnight at 4 ℃. On the next day, the secondary antibody was incubated at room temperature for 30 min, and then incubated with hyperactive protein A/G-Tn5 transposase for 30 min to obtain fragmented DNA. The DNA library was purified and amplified for sequencing with the Illumina NovaSeq 6000 platform.

For CUT&Tag data analysis, FastQC software was first used to evaluate the quality of the raw data and remove the poor quality data. Low quality reads and adapters were removed by trim-galore, using parameters ‘-q 20–length 20–stringency 3’, so all reads with MAPQ larger than 20 and longer than 20nt are kept for adapter removal and subsequent analysis. BWA or Bowtie software was applied to map the quality filtered data with the reference genome. MACS was available to perform peak calling for each sample, and then genomic site annotation, motif analysis, and GO and KEGG enrichment analysis of target genes were implemented on these peak regions. The IGV tool was employed to convert raw bam files to bigwig files, ensuring the read count data was visualized.

### Xenograft tumor model in Balb/c nude mice

Female Balb/c nude mice (4–5 weeks old) were purchased from the Department of Laboratory Animal Science, Peking University Health Science Center. They were raised in a specific pathogen-free environment with controlled temperature and humidity. Z138 cells were suspended in a mixture of PBS and Matrigel (PBS:Matrigel = 1:1), and each mouse was subcutaneously injected with 200 μl cell suspension containing 10^7^ cells into the right flank region to establish MCL model. Tumor size and mice body weight were measured every 2 days, and tumor volume was calculated with the formula V = length × width^2^/2^27^. When the tumor volume reached 100–120 mm^3^ (10 days after tumor implantation), the mice were randomly divided into the following 4 groups (n = 6 in each group) and the treatment started: control group (normal saline), nutlin-3a group (40 mg/kg), apcin group (20 mg/kg), and nutlin-3a (40 mg/kg) plus apcin (20 mg/kg) group. Normal saline, nutlin-3a and apcin were administrated intraperitoneally every other day for 2 weeks (a total of 7 doses). On the first day after the last treatment, 20 μl eyeball blood was collected from each mouse for blood routine test. On the second day after the last treatment, one eyeball of each mouse was removed to get the whole blood, and the serum was obtained by centrifugation for blood biochemistry test. Meanwhile, mice were sacrificed and their hearts, livers, spleens, lungs, kidneys and tumors were harvested for immunohistochemical (IHC) and hematoxylin–eosin (HE) staining. This animal experiment was approved by the Laboratory Animal Welfare Ethics Committee of Peking University Health Science Center (LA2016029).

### Immunohistochemiscal (IHC) and hematoxylin–eosin (HE) staining

Immunohistochemical (IHC) staining was performed on paraffin sections of MCL patients, LRH patients, and mice tumors. Sections were incubated with primary antibodies overnight at 4 ℃. CDC20 (1:500, 10252-1-AP, Proteintech, China) and cyclin D1 (1:1000, 26939-1-AP, Proteintech, China) primary antibodies were used to stain tissues from MCL and LRH patients, while p53 (1:3200, 60283-2-Ig, Proteintech, China), CDC20 (1:200, 10252-1-AP, Proteintech, China), cleaved PARP (1:200, #94885, CST, USA), and Ki-67 (1:400, #12202, CST, USA) primary antibodies were used to stain mice tumor tissues. All sections were scanned with a digital scanner (Aperio Versa 8, Leica, Germany) and images were presented and captured by Aperio ImageScope software (Leica, Germany). The targeted protein expression was analyzed quantitatively by mean optical density (MOD) [36]. Six images of each section at 200 × magnification were randomly captured, and the integrated optical density (IOD) and positive staining area of each image were measured by Image-Pro Plus software (Version 6.0, Media Cybernetics, USA). MOD of each image was acquired by dividing IOD by the positive staining area, and MOD of each section was the average of MOD of six images. The percentage of cyclin D1 positive cells in each MCL patient was calculated by HALO image analysis platform (Version 3.3.2541.345, Indica Labs, USA), defined as the proportion of positive staining cells to total cells on a scanning image.

In vivo safety of nutlin-3a and apcin was evaluated by hematoxylin–eosin (HE) staining of mice heart, liver and kidney tissue sections. Sections were routinely deparaffinized and hydrated. Then, they were soaked in Harris hematoxylin (BASO, Zhuhai, China) solution for 5 min to stain nuclei and soaked in eosin (BASO, Zhuhai, China) solution for 40 s to stain cytoplasm and extracellular matrix.

### Statistical analysis

SPSS software (Version25.0, IBM SPSS Inc., Chicago, IL, USA) was used for statistical analysis, and Graphpad Prism software (Version9.0.2, San Diego, CA, USA) was used to draw statistical graphs. Measurement data with normal distribution were presented as mean ± standard deviation (SD) and analyzed by independent t-test (2 groups) or one-way ANOVA (≥ 3 groups). Measurement data with skewed distribution were shown as median, maximum and minimum, and compared by Mann–Whitney U test. Counting data were expressed as number (n) and percentage (%) and evaluated by Chi-square test. For correlation analysis, R language (Version 4.1.2) was used for statistical analysis and drawing statistical charts. Statistical significance was defined as *P* < 0.05.

## Results

### CDC20 was upregulated in MCL, and was closely related to clinicopathological features and prognosis of MCL patients

**Fig. 1:**
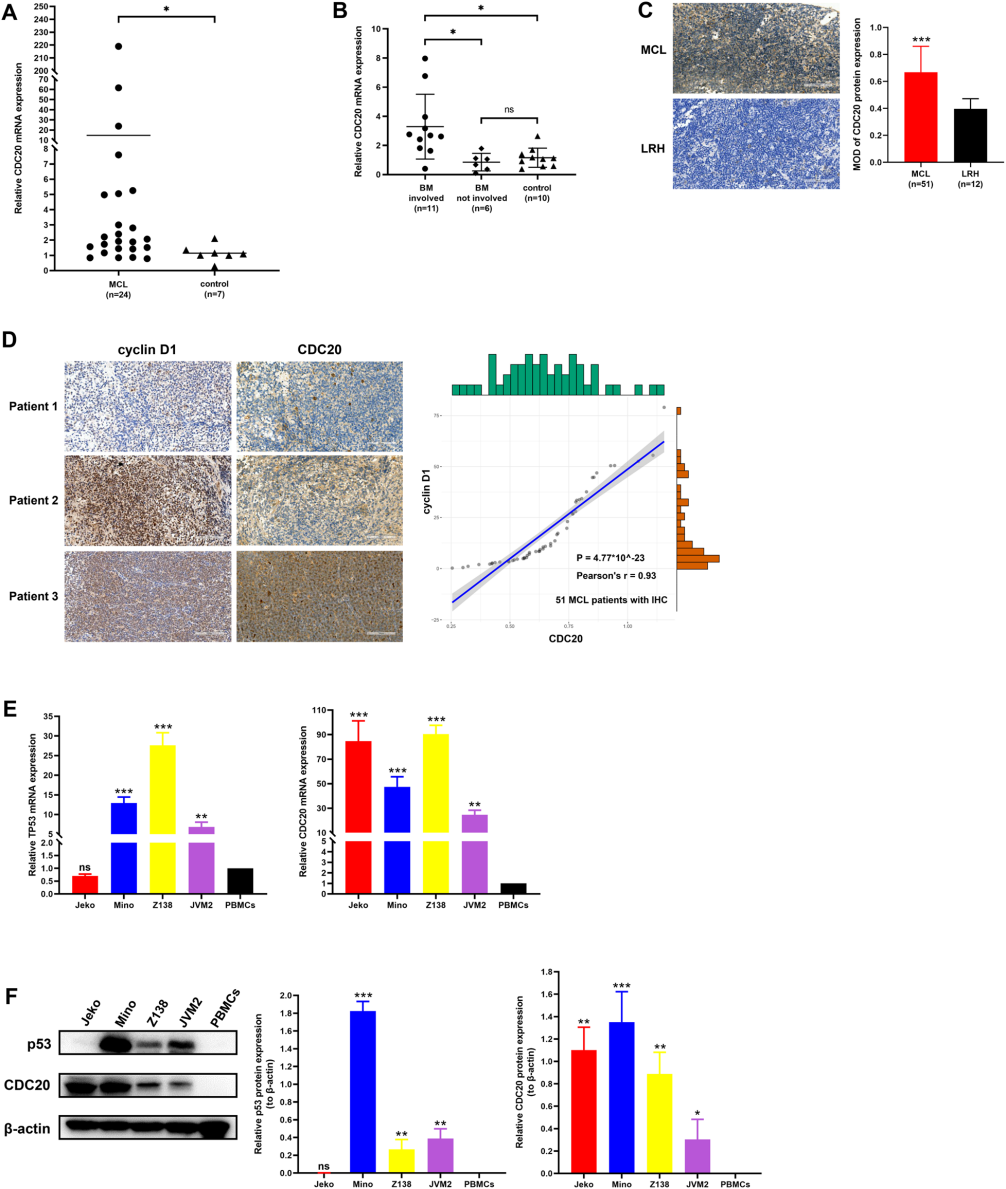
High expression of CDC20 in MCL patients and cell lines. **A** Relative CDC20 mRNA expression of PBMCs from 24 MCL patients and 7 healthy controls was detected by RT-qPCR. **B** Relative CDC20 mRNA expression of BMNCs from 17 MCL patients (including 11 cases with involved bone marrow and 6 cases with uninvolved bone marrow) and 10 healthy donors was detected by RT-qPCR. **C** Representative CDC20 IHC images of MCL patients and LRH patients (left, × 200 magnification; scale bar, 100 μm). Positive expression of CDC20 in 51 MCL patients and 12 LRH patients (as controls) was determined by MOD value (right). **D** Representative cyclin D1 and CDC20 IHC images of MCL patients (left, × 200 magnification; scale bar, 100 μm). The positive relationship between cyclin D1 expression and CDC20 expression was confirmed by IHC staining in 51 MCL patients (Right). **E** Relative TP53 and CDC20 mRNA expression of MCL cell lines Jeko, Mino, Z138 and JVM2 was detected by RT-qPCR, with PBMCs as control. **F** Relative p53 and CDC20 protein expression of MCL cell lines Jeko, Mino, Z138 and JVM2 was analyzed by WB, with PBMCs as control. *P < 0.05, **P < 0.01, ***P < 0.001, ns meant* P* > 0.05

We first verified the expression level of CDC20 in MCL patients and cell lines. CDC20 mRNA expression was detected by RT-qPCR in PBMCs and BMNCs of MCL patients and healthy controls, and CDC20 protein expression was assessed by IHC analysis of pathological sections of MCL and LRH patients. The results showed that CDC20 expression was significantly increased in PBMCs of MCL patients (n = 24) compared with healthy controls (n = 7) (Fig. 1A). There was no significant difference of CDC20 expression between bone marrow uninvolved MCL patients (n = 6) and healthy donors (n = 10), whereas CDC20 was overexpressed in MCL patients with bone marrow involvement (n = 11) (Fig. 1B). Similarly, IHC staining of CDC20 quantified by MOD revealed that CDC20 protein expression of MCL patients (n = 51) was higher than that of LRH patients (n = 12) (Fig. 1C). Moreover, we found the percentage of cyclin D1 positive cells, a typical IHC marker of MCL, was positively correlated with CDC20 expression level (Fig. 1D). For MCL cell lines, CDC20 expression was significantly upregulated in all the four cell lines at mRNA (Fig. 1E) and protein (Fig. 1F) levels compared with healthy PBMCs, and CDC20 expression of p53-mutant cells Jeko and Mino was higher than that of p53-wild type cells Z138 and JVM2 (Fig. 1F).

**Fig. 2:**
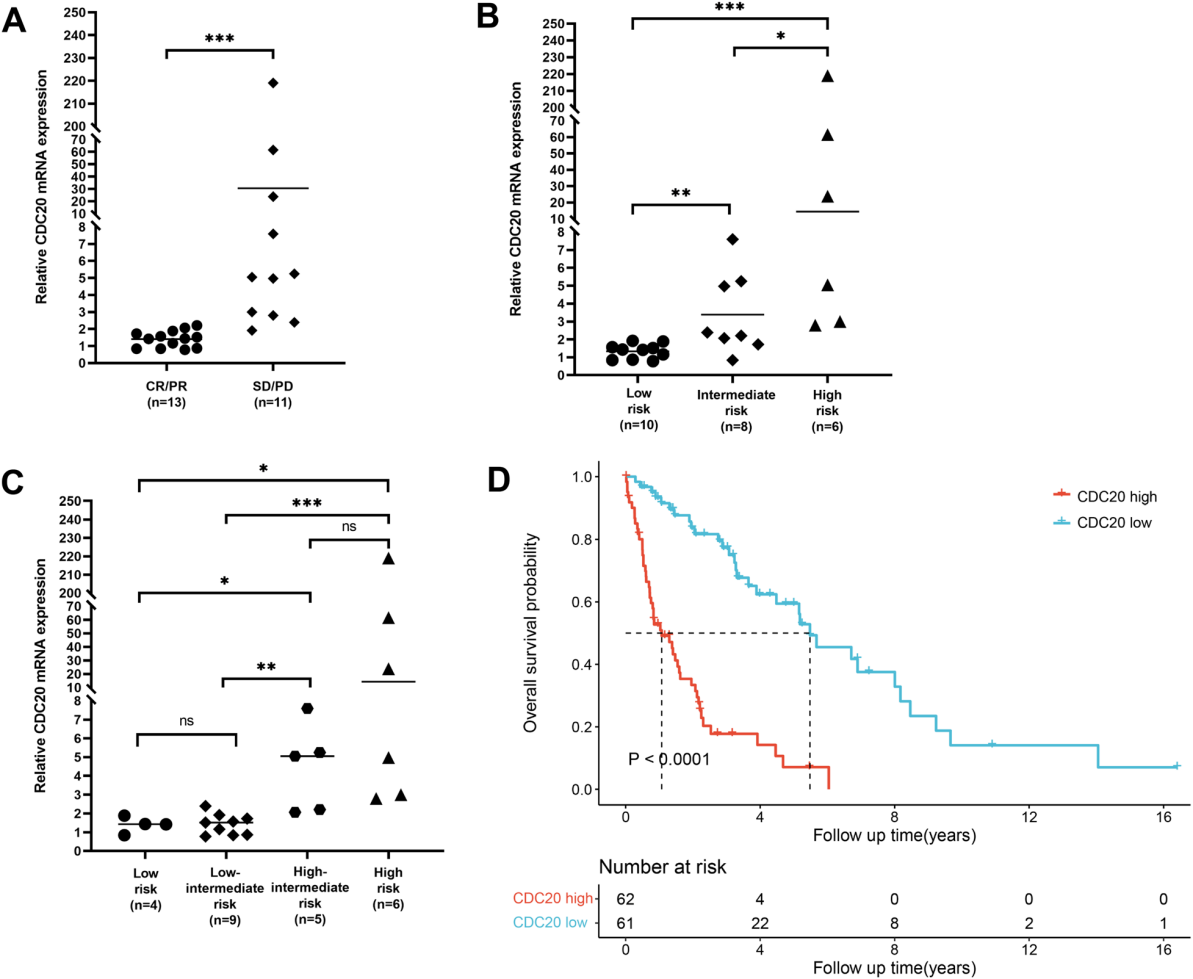
CDC20 mRNA expression was associated with the therapeutic effect and prognosis of MCL patients. Among 24 MCL patients with PBMCs extracted, CDC20 mRNA expression was significantly different in the subgroups of treatment response (**A**), MIPI score (**B**), and MIPI-c score (**C**). **A** Patients were divided into the CR/PR group and the PD/SD group according to treatment response, and CDC20 mRNA expression level between the two groups was compared. **B** Patients were divided into the low risk group, the intermediate risk group and the high risk group according to MIPI score, and CDC20 mRNA expression level among the three groups was compared. **C** Patients were divided into the low risk group, low-intermediate risk group, high-intermediate risk group, and the high risk group according to MIPI-c score, and CDC20 mRNA expression level among the four groups was compared. **D** The relationship between CDC20 expression level and overall survival of MCL patients was analyzed via GSE93291 dataset (n = 123). *P < 0.05, **P < 0.01, ***P < 0.001, ns meant *P* > 0.05

The association between CDC20 mRNA expression level and clinicopathological parameters of MCL patients was further evaluated. In 24 MCL patients with PBMCs extracted, subgroup analysis was performed on treatment response, MCL International Prognostic Index (MIPI) score and combined MCL International Prognostic Index (MIPI-c) score. According to treatment response, MCL patients were divided into the complete remission (CR)/partial remission (PR) group (n = 13) and the stable disease (SD)/progressive disease (PD) group (n = 11). The results suggested that CDC20 expression in the CR/PR group was significantly lower than that in the SD/PD group (Fig. 2A). According to MIPI score, MCL patients was divided into the low risk group (n = 10), the intermediate risk group (n = 8) and the high risk group (n = 6), and the results showed that the higher the risk level, the higher the expression of CDC20 (Fig. 2B). Similar results were also found in the MIPI-c score-based grouping, which classified MCL patients into the low risk group (n = 4), the low-intermediate risk group (n = 9), the high-intermediate risk group (n = 5), and the high risk group (n = 6). The results indicated that CDC20 expression in the low and low-intermediate risk patients was significantly lower than that in the high-intermediate risk and high risk patients (Fig. 2C). Furthermore, the relationship between CDC20 protein expression level and clinicopathological features in 51 MCL patients with CDC20 IHC staining was also analyzed. As shown in Table 2, CDC20 expression level was significantly correlated with Ki-67 expression (P = 0.014), LDH serum level (P = 0.007), tumor stage (P = 0.040), MIPI score (P = 0.008), number of involved lymph node areas (P = 0.036), bone marrow involvement (P = 0.015) and treatment response (P = 0.002), but was not significantly associated with age, gender, B symptoms, pathological type, ECOG score, white blood cell count and β2-MG serum level.

**Table 2:** Correlation between CDC20 expression and clinical features in 51 MCL patients

Clinical features	Cases, N	CDC20 expression		χ2	P value
		Low, n (%)	High, n (%)		
Total	51	21 (41.2)	30 (58.8)		
Age					
<60	21	9 (42.9)	12 (57.1)	0.042	0.838
≥ 60	30	12 (40.0)	18 (60.0)		
Gender					
Male	39	15 (38.5)	24 (61.5)	0.140	0.708
Female	12	6 (50.0)	6 (50.0)		
B symptoms					
Yes	24	8 (33.3)	16 (66.7)	1.151	0.283
No	27	13 (48.1)	14 (51.9)		
Pathological type					
Indolent	9	5 (55.6)	4 (44.4)	0.351	0.553
Aggressive	42	16 (38.1)	26 (61.9)		
ECOG					
<2	45	20 (44.4)	25 (55.6)	0.735	0.391
≥ 2	6	1 (16.7)	5 (83.3)		
Ki-67					
<30%	19	12 (63.2)	7 (36.8)	6.041	0.014*
≥ 30%	32	9 (28.1)	23 (71.9)		
WBC					
<10*10^9/L	34	13 (38.2)	21 (61.8)	0.364	0.546
≥ 10*10^9/L	17	8 (47.1)	9 (52.9)		
β2-MG					
<3 mg/L	21	11 (52.4)	10 (47.6)	1.850	0.174
≥ 3 mg/L	30	10 (33.3)	20 (66.7)		
LDH					
<240 IU/L	30	17 (56.7)	13 (43.3)	7.217	0.007**
≥ 240 IU/L	21	4 (19.0)	17 (81.0)		
Stage					
I-II	11	8 (72.7)	3 (27.3)	4.223	0.040*
III-IV	40	13 (32.5)	27 (67.5)		
MIPI					
Low risk	20	13 (65.0)	7 (35.0)	9.743	0.008**
Intermediate risk	12	5 (41.7)	7 (58.3)		
High risk	19	3 (15.8)	16 (84.2)		
Involved lymph node areas					
<3	16	10 (62.5)	6 (37.5)	4.377	0.036*
≥ 3	35	11 (31.4)	24 (68.6)		
BM involvement					
Yes	41	13 (31.7)	28 (68.3)	5.875	0.015*
No	10	8 (80.0)	2 (20.0)		
Treatment response					
CR/PR	31	18 (58.1)	13(41.9)	9.308	0.002**
SD/PD	20	3 (15.0)	17 (85.0)		

*ECOG* Eastern Cooperative Oncology Group, *WBC* white blood cell, *LDH* lactate dehydrogenase, *β2-MG* β2-microglobulin, *MIPI* MCL International Prognostic Index, *BM* bone marrow, *CR* complete remission, *PR* partial remission, *SD* stable disease, *PD* progressive disease

Bold values meant *P < 0.05 or **P ＜0.01.

Whether CDC20 expression could be served as a prognostic factor of MCL patients was also investigated. Overall survival (OS) was analyzed on GSE93291 dataset (n = 123) from GEO database, and the result implied that MCL patients with high CDC20 expression had shorter OS (Fig. 2D), suggesting the prognostic value of CDC20 in MCL patients.

### CDC20 inhibition could impede cell proliferation, migration and invasion, and induce apoptosis and cell cycle arrest

**Fig. 3:**
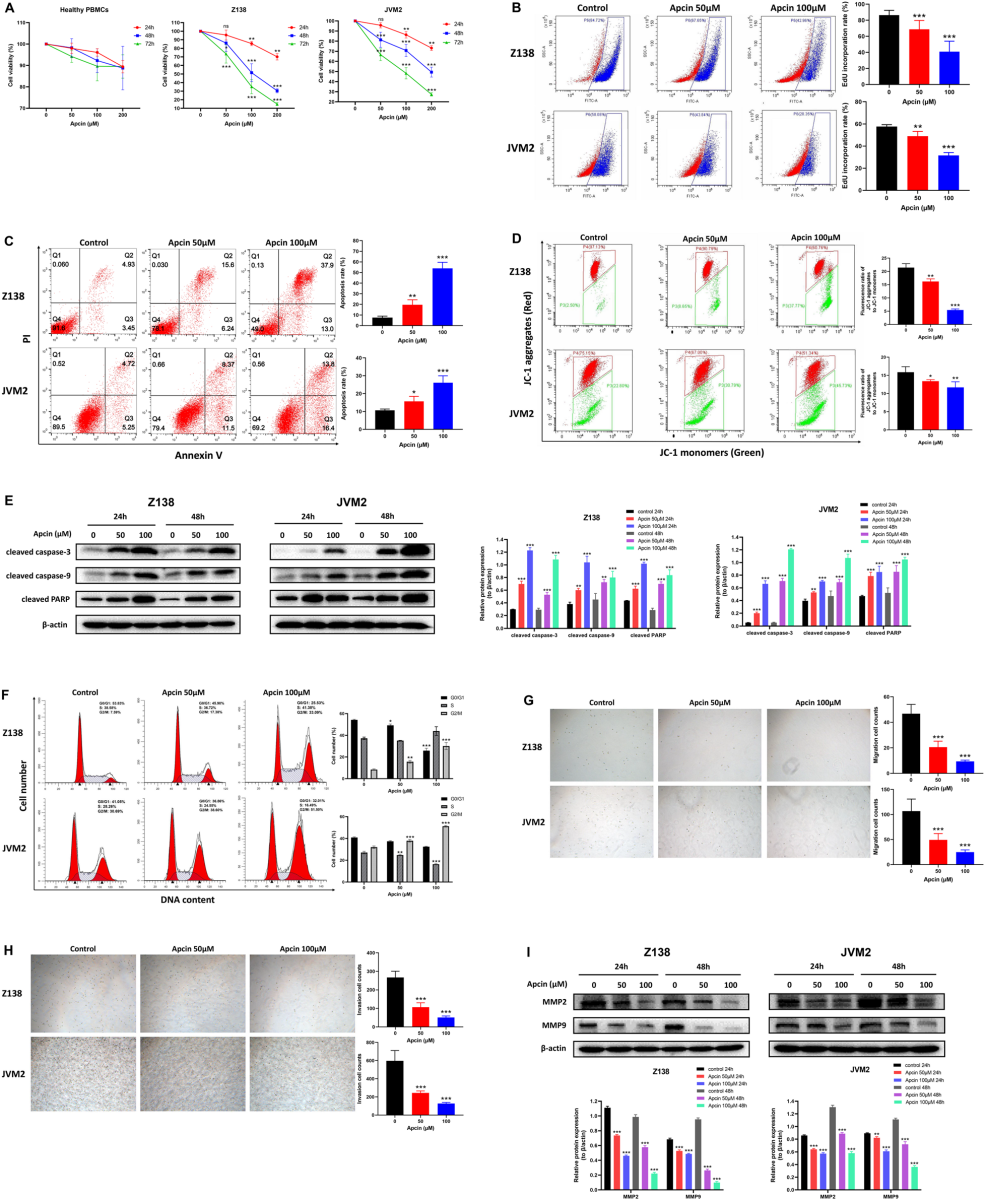
CDC20 inhibitor apcin could inhibit cell proliferation, migration and invasion, and induce cell apoptosis and cell cycle arrest in Z138 and JVM2 cells. **A** After healthy PBMCs, Z138 and JVM2 cells exposed to 50 μM, 100 μM and 200 μM apcin for 24 h, 48 h and 72 h, cell viability was assessed by CCK-8 assay. Cell viability at each time point was defined as the percentage obtained by dividing the OD value of the treatement group by the OD value of the corresponding untreated group at 450 nm. **B** After Z138 and JVM2 cells treated with 50 μM and 100 μM apcin for 48 h, EdU incorporation rate was detected by flow cytometry to determine the cell proliferation condition. **C** The apoptosis rate was measured by flow cytometry based on Annexin V-FITC/PI staining after Z138 and JVM2 cells treated with 50 μM and 100 μM apcin for 48 h. The total apoptosis rate was the sum of the early apoptosis rate (right lower quadrant) and the late apoptosis rate (right upper quadrant). **D** The changes of MMP was estimated by flow cytometry based on JC-1 fluorescent probe after Z138 and JVM2 cells incubated with 50 μM and 100 μM apcin for 48 h. The results were presented as the ratio of mean red fluorescence intensity to mean green fluorescence intensity. Decreased ratio indicated the decrease in MMP, which was also a hallmark event of early apoptosis. **E** Expression of Apoptosis-related proteins by WB analysis after 24 h and 48 h apcin treatment in Z138 and JVM2 cells. **F** The proportion of G0/G1, S and G2/M phases in the cell cycle was analyzed by PI flow cytometry after Z138 and JVM2 cells treated with 50 μM and 100μmM apcin for 48 h (Z138) or 72 h (JVM2). **G**, **H** The effect of apcin on cell migration (**G**) and invasion (**H**) was confirmed by Transwell assays after Z138 and JVM2 cells exposed to 50 μM and 100 μM apcin for 48 h. Images were captured by an inverted microscope (× 10 magnification). (**I**) Migration and invasion-related proteins was analyzed by WB after 24 h and 48 h apcin treatment in Z138 and JVM2 cells. The above data were obtained from at least three independent experiments and presented as mean ± SD. *P < 0.05, **P < 0.01, ***P < 0.001 compared with the control group

Apcin, an inhibitor of APC/C-CDC20 [37], was used to demonstrate the effect of CDC20 on the cell phenotype of MCL cell lines. The CCK-8 assay was used to test cell viability of healthy PBMCs, Z138 and JVM2 cells treated with designated concentration of apcin (0, 50 μM, 100 μM, 200 μM) for 24 h, 48 h and 72 h. The results showed significant decrease in cell viability of both Z138 and JVM2 cells in a dose- and time-dependent manner, while apcin-treatment almost had no effect on healthy PBMCs (Fig. 3A). Correspondingly, EdU cell proliferation assay also proved that the higher the apcin concentration, the lower the EdU incorporation rate in Z138 and JVM2 cells after 48 h apcin exposure (Fig. 3B). These results confirmed that CDC20 repression inhibited MCL cell growth.

Apoptotic cells were analyzed by flow cytometry after Z138 and JVM2 cells treated with apcin for 48 h. Compared with the control group, both apcin-treatment Z138 and JVM2 cells underwent obvious apoptosis, and the percentage of apoptotic cells increased in a dose-dependent manner (Fig. 3C). Further, JC-1 fluorescent probe was used to detect the changes of mitochondrial membrane potential (MMP) in MCL cells. As shown in Fig. 3D, the fluorescence ratio in Z138 and JVM2 cells reduced with the increase of apcin concentration, confirming that suppressed CDC20 led to early cell apoptosis. Apoptosis-related proteins were also examined by WB, and the results implied that cleaved caspase-3, cleaved caspase-9 and cleaved PARP all increased after Z138 and JVM2 cells exposed to 50 μM and 100 μM apcin for 24 h and 48 h (Fig. 3E).

Cell cycle assay was performed to observe whether CDC20 had certain effect on the MCL cell cycle. Both Z138 and JVM2 cells were found to be arrested at the G2/M phase after treatment with 50 μM and 100 μM apcin for 48 h in Z138 cells or 72 h in JVM2 cells. In addition, G0/G1 phase fraction was decreased in Z138 cells, while S phase fraction was depressed in JVM2 cells (Fig. 3F). These findings suggested that downregulation of CDC20 mainly resulted in G2/M arrest in the MCL cell cycle.

Cell migration and invasion abilities with CDC20 inhibition were further examined based on Transwell assays. The number of migratory and invasive Z138 and JVM2 cells in the apcin-treatment group was significantly lower than that in their corresponding control group in a concentration-dependent manner (Fig. 3G and H). Not surprisingly, the protein expression of MMP2 and MMP9, two factors regulating cell migration and invasion, decreased after exposed to apcin in Z138 and JVM2 cells (Fig. 3I). Therefore, low expression of CDC20 could effectively inhibit cell motilities.

### Activated p53 downregulated CDC20 expression, and the effect of p53 activation on MCL cell phenotype was similar to CDC20 inhibition

**Fig. 4:**
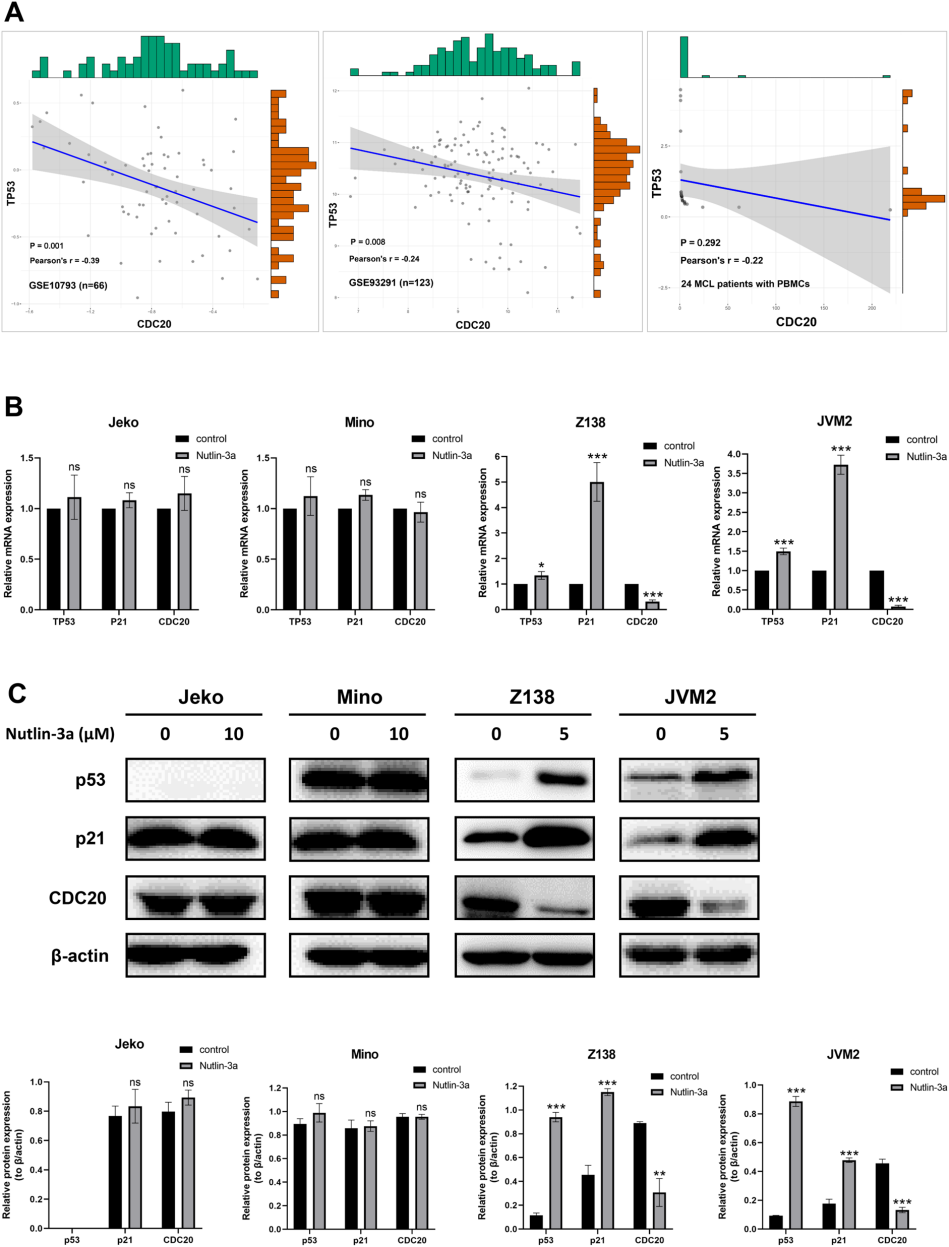
p53 activation could downregulate CDC20 expression. **A** GSE10793 (n = 66), GSE93291 (n = 123) and patients in our cohort (n = 24) showed that TP53 expression was negatively correlated with CDC20 expression. **B** The mRNA expression level of TP53, P21 and CDC20 in Jeko, Mino, Z138 and JVM2 cells were detected by RT-qPCR after treatment with 5 μM (Z138 and JVM2) or 10 μM (Jeko and Mino) nutlin-3a for 24 h. **C** The protein expression level of p53, p21 and CDC20 in Jeko, Mino, Z138 and JVM2 cells were analyzed by WB after treatment with 5 μM (Z138 and JVM2) or 10 μM (Jeko and Mino) nutlin-3a for 24 h. The increase in p53 expression was accompanied by a decrease in CDC20 expression only in Z138 and JVM2 cells (**B**, **C**). Data were obtained from at least three independent experiments and presented as mean ± SD. *P < 0.05, ***P < 0.001 compared with the control group, ns meant *P* > 0.05

To clarify the relationship between p53 and CDC20 in MCL, we first analyzed the correlation between p53 and CDC20 via GSE10793 (n = 66) and GSE93291 (n = 123) datasets of MCL patients and 24 MCL patients with PBMCs extracted in our study. As shown in Fig. 4A, the expression level of p53 was negatively correlated with the expression level of CDC20 in GSE10793 and GSE93291 datasets. There was a negative relationship between p53 and CDC20 in our patient cohort, but it had no statistical significance. Besides, RT-qPCR and WB results validated that the mRNA and protein expression level of CDC20 were significantly reduced after p53 activation in p53-wild type Z138 and JVM2 cells treated with nutlin-3a for 24 h, while the expression level of CDC20 had no significant change in p53-mutant Jeko and Mino cells after nutlin-3a exposure (Fig. 4B and C). Induction of p21 expression was a hallmark of p53 activation since p21 was a defined downstream target of p53. In Z138 and JVM2 cells, p21 was accumulated with nutlin-3a treatment, whereas this phenomenon was not observed in Jeko and Mino cells (Fig. 4B and C). Collectively, these data illustrated that CDC20 expression was downregulated by p53 in MCL, and activated status of p53 was necessary for CDC20 regulation.

**Fig. 5:**
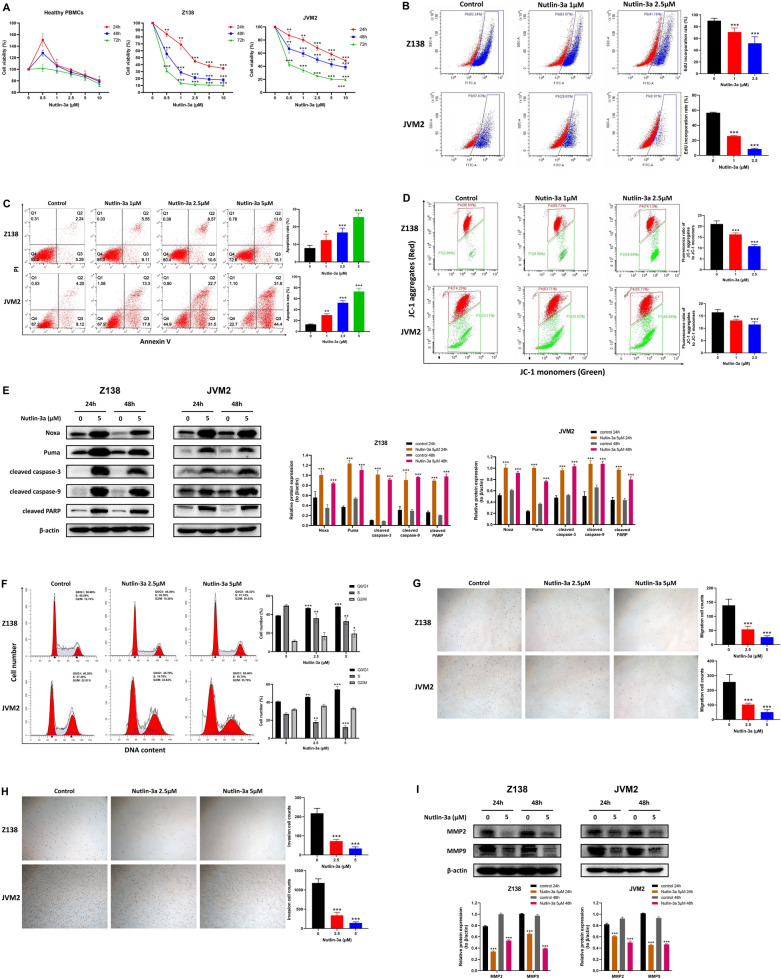
Nutlin-3a treatment led to attenuated cell proliferation, migration and invasion as well as enhanced apoptosis and cell cycle arrest in Z138 and JVM2 cells. **A** After healthy PBMCs, Z138 and JVM2 cells exposed to 0.5 μM, 1 μM, 2.5 μM, 5 μM and 10 μM nutlin-3a for 24 h, 48 h and 72 h, cell viability was assessed by CCK-8 assay. **B** After Z138 and JVM2 cells treated with 1 μM and 2.5 μM nutlin-3a for 48 h, EdU incorporation rate was detected by flow cytometry to determine the cell proliferation condition. **C** After Z138 and JVM2 cells treated with 1 μM, 2.5 μM and 5 μM nutlin-3a for 48 h, the apoptosis rate was quantified by flow cytometry based on Annexin V-FITC/PI staining. **D** The changes of MMP in Z138 and JVM2 cells was examined by flow cytometry based on JC-1 fluorescent probe after exposed to 1 μM and 2.5 μM nutlin-3a for 48 h. **E** Targeted protein expression by WB analysis after 24 h and 48 h 5 μM nutlin-3a treatment in Z138 and JVM2 cells. **F** The proportion of G0/G1, S and G2/M phases in the cell cycle was analyzed by PI flow cytometry after Z138 and JVM2 cells treated with 2.5 μM and 5 μM nutlin-3a for 48 h (Z138) or 72 h (JVM2). **G**, **H** After treatment with 2.5 μM and 5 μM nutlin-3a for 48 h in Z138 and JVM2 cells, the effect of nutlin-3a on cell migration (**G**) and invasion (**H**) was confirmed by Transwell assays. Images were captured by an inverted microscope (× 10 magnification). **I** Migration and invasion-related proteins were analyzed by WB after 24 h and 48 h nutlin-3a treatment in Z138 and JVM2 cells. The above data were obtained from at least three independent experiments and presented as mean ± SD. *P < 0.05, **P < 0.01, ***P < 0.001 compared with the control group

We wondered whether p53 activation could achieve similar effects on MCL biological behaviors as those provoked by CDC20 suppression. The CCK-8 results showed nutlin-3a could inhibit cell growth in a dose- and time-dependent manner in Z138 and JVM2 cells, while its effect on healthy PBMCs was negligible (Fig. 5A). EdU cell proliferation assay uncovered that 48 h nutlin-3a treatment led to a significant reduction of EdU incorporation in Z138 and JVM2 cells (Fig. 5B), which was accompanied with a concentration-dependent cell apoptosis induction (Fig. 5C). JC-1 test also indicated that the fluorescence ratio decreased with the increase of nutlin-3a concentration in Z138 and JVM2 cells (Fig. 5D). Moreover, increased protein expression of Noxa, Puma, cleaved caspase-3, cleaved caspase-9 and cleaved PARP was found after Z138 and JVM2 cells treated with 5 μM nutlin-3a for 24 h and 48 h (Fig. 5E). Noxa and Puma were p53-regulated pro-apoptotic proteins. Thus, p53 activation in MCL cells could hinder cell proliferation and lead to apoptotic events.

Cell cycle was analyzed after Z138 and JVM2 cell incubated with nutlin-3a for 48 h. As Fig. 5F presented, the accumulation in G0/G1 phase as well as the reduction in S phase were observed in both Z138 and JVM2 cells in a dose-dependent manner. Additionally, G2/M arrest was found in Z138 cells after treatment with higher dose of nutlin-3a (5 μM). These findings clarified that p53 activation brought about abnormal cell cycle progression, characterized by increased G0/G1 and G2/M phase fraction and reduced S phase fraction.

The impact of nutlin-3a treatment on cell motilities was evaluated. Expectedly, the abilities of cell migration and invasion was significantly attenuated (Fig. 5G and H) with the decreased expression of MMP2 and MMP9 proteins (Fig. 5I) in Z138 and JVM2 cells treated with nutlin-3a compared with the control group, further proving that p53 activation had inhibitory effect on MCL cell motilities.

### p53 negatively regulated CDC20 expression through directly binding to CDC20 promoter

We have demonstrated that p53 activation suppressed the expression of CDC20 at the mRNA and protein level. Activating p53 or inhibiting CDC20 exerted the same effects on MCL cells, including cell proliferation inhibition, apoptosis induction, cell cycle arrest, and impaired migratory and invasive capabilities. Considering that p53 was a fundamental transcription factor, we further investigated the mechanism of how p53 transcriptionally regulating CDC20 in MCL.

**Fig. 6:**
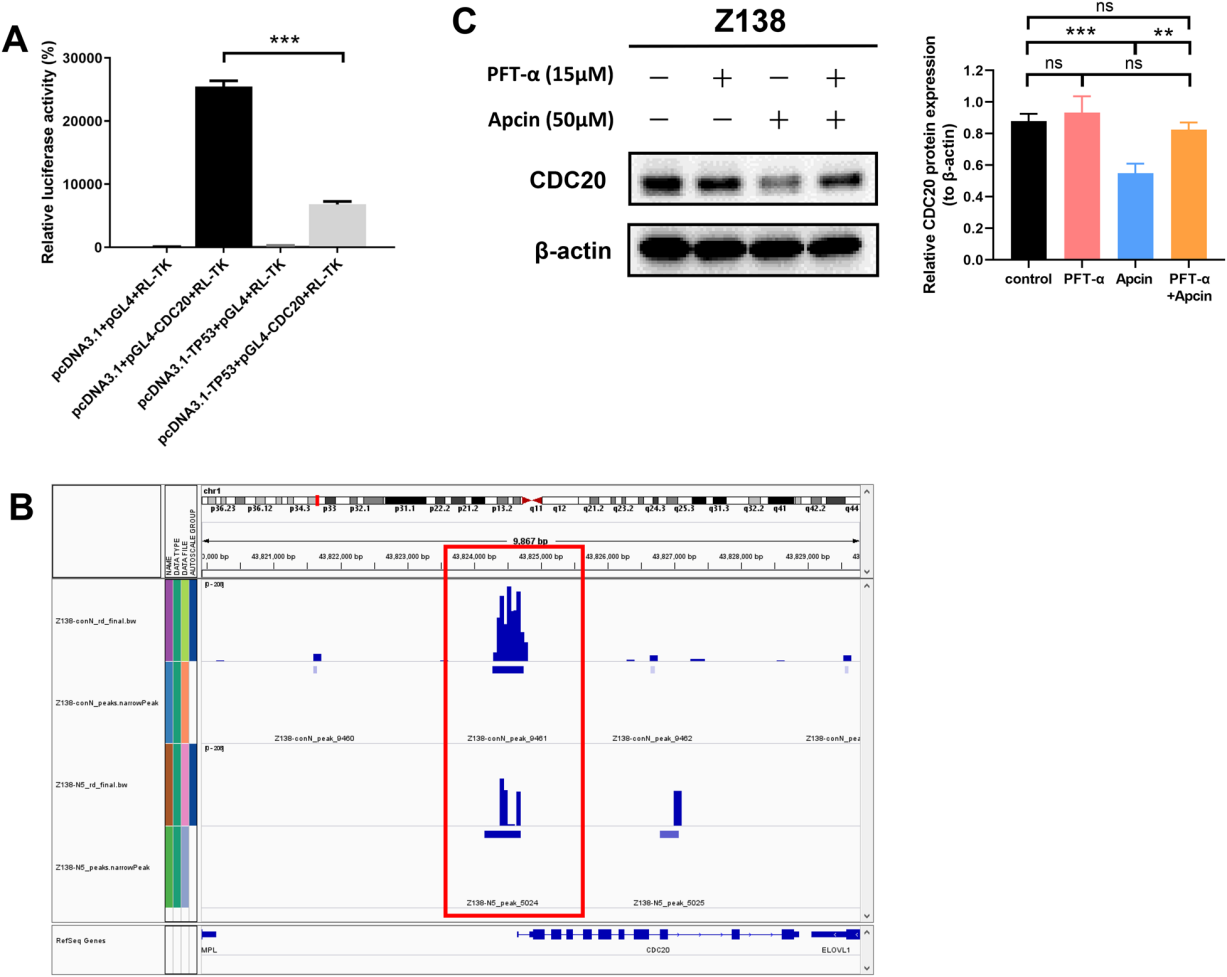
p53 transcriptionally repressed CDC20 expression by directly binding to CDC20 promoter. **A** Dual-luciferase reporter gene assay was conducted to verify whether p53 could regulate CDC20 promoter activity (TSS-1495 bp ~  + 26 bp). The results implied that p53 overexpression in 293 T cells could significantly inhibit CDC20 promoter activity. Relative luciferase activity was expressed as the ratio of firefly luciferase activity to *renilla* luciferase activity. Data were obtained from three independent experiments and presented as mean ± SD. ***P < 0.001. **B** CUT&Tag assay proved that p53 directly bound to CDC20 promoter region at TSS-492 bp ~  + 101 bp. Compared with the untreated control group, CDC20 promoter signal was significantly decreased in Z138 cells treated with 5 μM nutlin-3a for 24 h. The red box indicated the binding site of p53 on CDC20 promoter on chromosome 1, and the characteristic signal peaks of the control group (upper) and nutlin3a-treated group (lower) in this region. **C** Z138 cells were pre-treated with 15 μM PFT-α for 1 h, and then co-treated with 50 μM apcin for 24 h. WB results showed pre-treatment of Z138 cells with PFT-α could rescue CDC20 expression reduced by apcin treatment. Data were obtained from at least three independent experiments and presented as mean ± SD. **P < 0.01, ***P < 0.001, ns meant *P* > 0.05

Dual-luciferase reporter gene assay was performed in 293 T cells to discuss the effect of p53 on CDC20 promoter activity. As displayed in Fig. 6A, after co-transfection with pcDNA3.1-TP53 and pGL4-CDC20, the relative luciferase activity was significantly reduced compared with that of single transfection with PGL4-CDC20, implying that p53 transcriptionally repressed CDC20 promoter activity in 293 T cells. CUT&Tag assay was implemented to further elucidate whether CDC20 was directly regulated by p53 in MCL cells. Untreated and nutlin-3a-treated Z138 cells were harvested and sequentially incubated with p53 primary antibody, secondary antibody and protein A/G-Tn5 transposase for DNA library construction and high-throughput sequencing. The results validated that p53 could directly bind to the promoter region of CDC20, and the binding site was located from 492 bp upstream to 101 bp downstream of TSS. The peak signal of CDC20 promoter was significantly lower in nutlin-3a-treated Z138 cells than that in the untreated group (Fig. 6B). Besides, pre-treatment of Z138 cells with 15 μM PFT-α, an inhibitor that inhibited p53-dependent transcriptional activity, could significantly increase CDC20 expression reduced by apcin treatment (Fig. 6C). Together, these data proved that p53 transcriptionally inhibited CDC20 expression by directly binding to CDC20 promoter.

### Combination of p53 activation and CDC20 inhibition had augmented anti-MCL activity

**Fig. 7:**
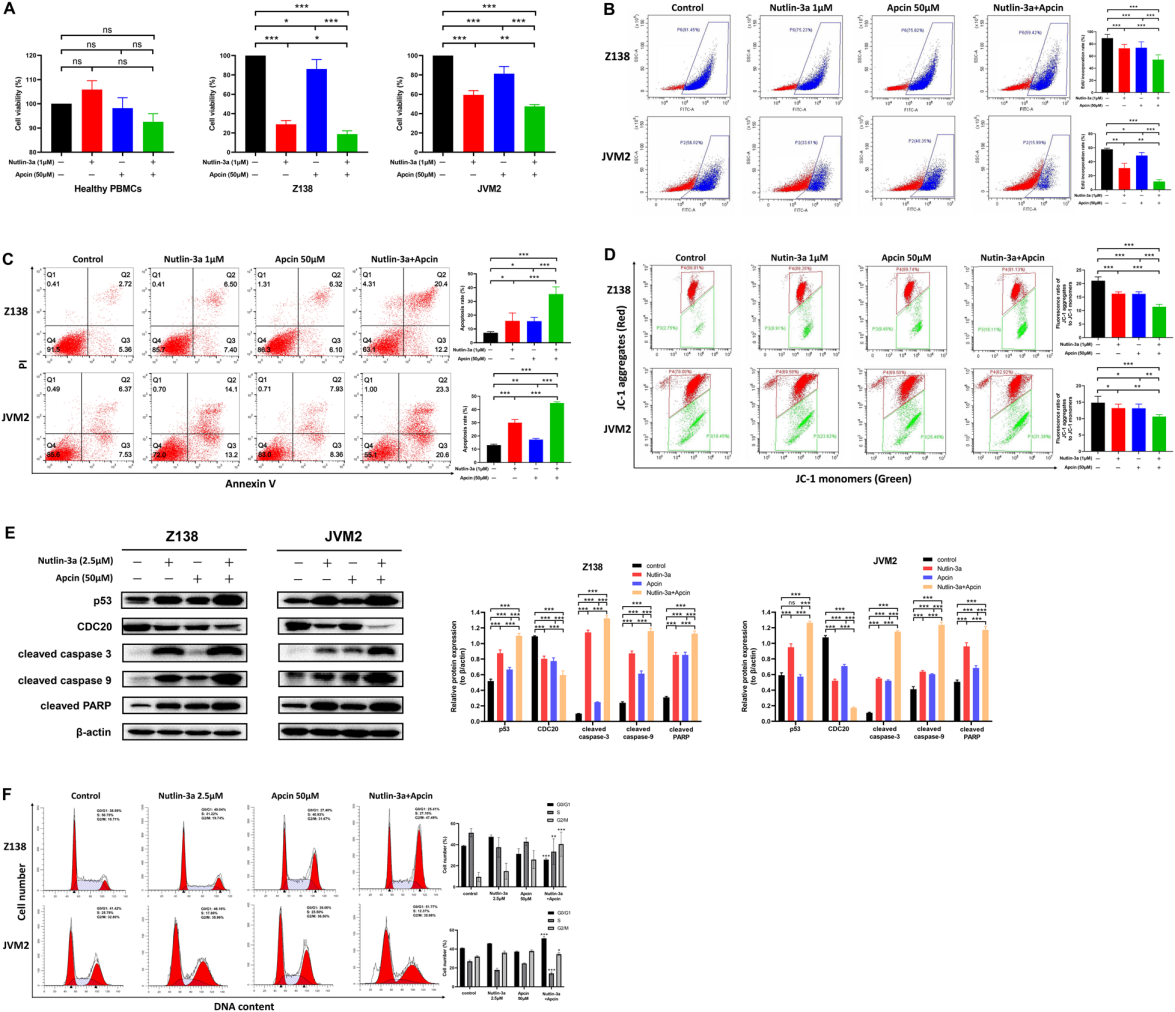
Enhanced effect on cell proliferation inhibition, apoptosis induction and cell cycle arrest after co-treatment with nutlin-3a and apcin in Z138 and JVM2 cells. **A** After healthy PBMCs, Z138 and JVM2 cells co-treated with 1 μM nutlin-3a and 50 μM apcin for 48 h, cell viability was assessed by CCK-8 assay. **B** After Z138 and JVM2 cells co-treated with 1 μM nutlin-3a and 50 μM apcin for 48 h, the EdU incorporation rate was tested by flow cytometry to determine the cell proliferation condition. **C** Z138 and JVM2 cells were treated with 1 μM nutlin-3a and 50 μM apcin in combination for 48 h, and the apoptosis rate was quantified by flow cytometry based on Annexin V-FITC/PI staining. **D** After co-treatment with 1 μM nutlin-3a and 50 μM for 48 h, the changes of MMP in Z138 and JVM2 cells were detected by flow cytometry based on the JC-1 fluorescent probe. **E** Targeted protein expression by WB analysis after 48 h co-treatment with 2.5 μM nutlin-3a and 50 μM apcin in Z138 and JVM2 cells. The above data were obtained from at least three independent experiments and presented as mean ± SD. *P < 0.05, **P < 0.01, ***P < 0.001. **F** After Z138 and JVM2 cells co-treated with 2.5 μM nutlin-3a and 50 μM apcin for 48 h (Z138) or 72 h (JVM2), the proportion of G0/G1, S and G2/M phases in the cell cycle was analyzed by PI flow cytometry. Data were obtained from at least three independent experiments and presented as mean ± SD. *P < 0.05, **P < 0.01, ***P < 0.001 compared with the control group

Considering the significant role of both p53 and CDC20 in MCL development and progression, the combined effect of nutlin-3a and apcin in Z138 and JVM2 cells was researched. As determined by CCK-8 assay, cell viability was greatly reduced in the combination group compared with nutlin-3a or apcin treated alone in Z138 and JVM2 cells, while it was not influenced by combinatory use or single use in healthy PBMCs (Fig. 7A). The CI values analyzed by CompuSyn revealed that combination of nutlin-3a and apcin with different concentrations had synergistic effect on Z138 and JVM2 cells (Table 3). EdU test showed that combinatory use of 1 μM nutlin-3a and 50 μM apcin exerted stronger tumor proliferation inhibition than single use (Fig. 7B). Apoptosis analysis suggested combination of 1 μM nutlin-3a and 50 μM apcin for 48 h caused marked cell apoptosis compared to the treatment with nutlin-3a or apcin alone (Fig. 7C). JC-1 assay exhibited the combination group had lower fluorescence ratio than the single treatment group (Fig. 7D). Further, compared with the single nutlin-3a or apcin group, we found the CDC20 protein expression was lowest and the protein expression of cleaved caspase-3, cleaved caspase-9 and cleaved PARP was highest in the combination group (Fig. 7E). In brief, combination of nutlin-3a and apcin repressed cell proliferation and promoted apoptosis more apparently than single reagent.

**Table 3:** Combination index (CI) values of Z138 and JVM2 cells treated with the combination of nutlin-3a and apcin for 48 h

Nutlin-3a (μM)	Apcin (μM)	CI (Z138)	CI (JVM2)
0.5	50	0.82	0.50
1	50	0.56	0.51
2.5	50	0.69	0.79
0.5	75	0.65	0.57
1	75	0.66	0.59
2.5	75	0.70	0.59
0.5	100	0.62	0.63
1	100	0.76	0.71
2.5	100	0.78	0.88

Furthermore, whether co-treatment with nutlin-3a and apcin had profound effect on cell cycle was evaluated. As shown in Fig. 7F, after co-treatment with nutlin-3a and apcin for 48 h, the proportion of G0/G1 phase and S phase was significantly reduced, while cell number in G2/M phase was significantly increased in Z138 cells. For JVM2 cells, combined treatment for 72 h resulted in cell accumulation in G0/G1 and G2/M phases, while the cell number in S phase was significantly reduced. The results suggested that combined effect of p53 activation and CDC20 inhibition on cell cycle was manifested by a reduction of S phase fraction and cell arrest in G2/M phase.

### Nutlin-3a/apcin alone or in combination could exert anti-tumor effect in vivo safely

**Fig. 8:**
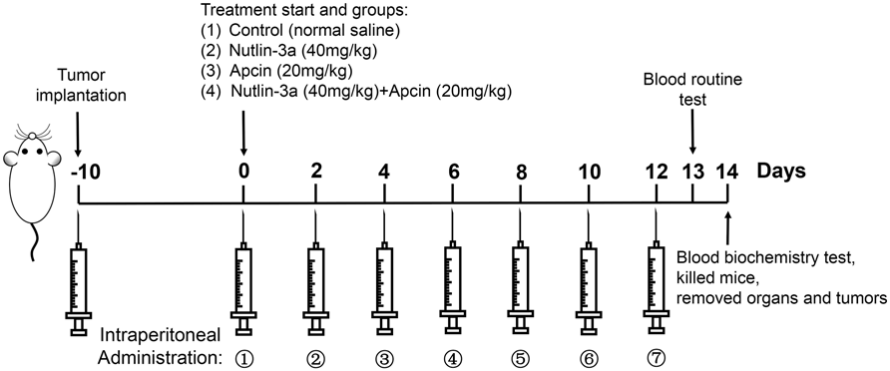
Diagram of the animal experiment

In vitro experiments have confirmed the potency of nutlin-3a and apcin to suppress MCL tumorigenesis. Whether nutlin-3a and apcin could have anti-tumor effect in vivo was determined. Human xenograft MCL model was established by subcutaneously injected Z138 cells into the right flank region of each female Balb/c nude mouse. The mice were randomly divided into 4 groups (n = 6 for each group) before administration, including the control group (normal saline, i.p.), the nutlin-3a group (40 mg/kg, i.p.), the apcin group (20 mg/kg, i.p.), and the nutlin-3a (40 mg/kg, i.p.) plus apcin (20 mg/kg, i.p.) group. Administration frequency was intraperitoneal injection (i.p.) every other day for 2 weeks (a total of 7 doses). All the 24 mice survived until they were sacrificed. The whole animal experiment was summarized in Fig. 8.

**Fig. 9:**
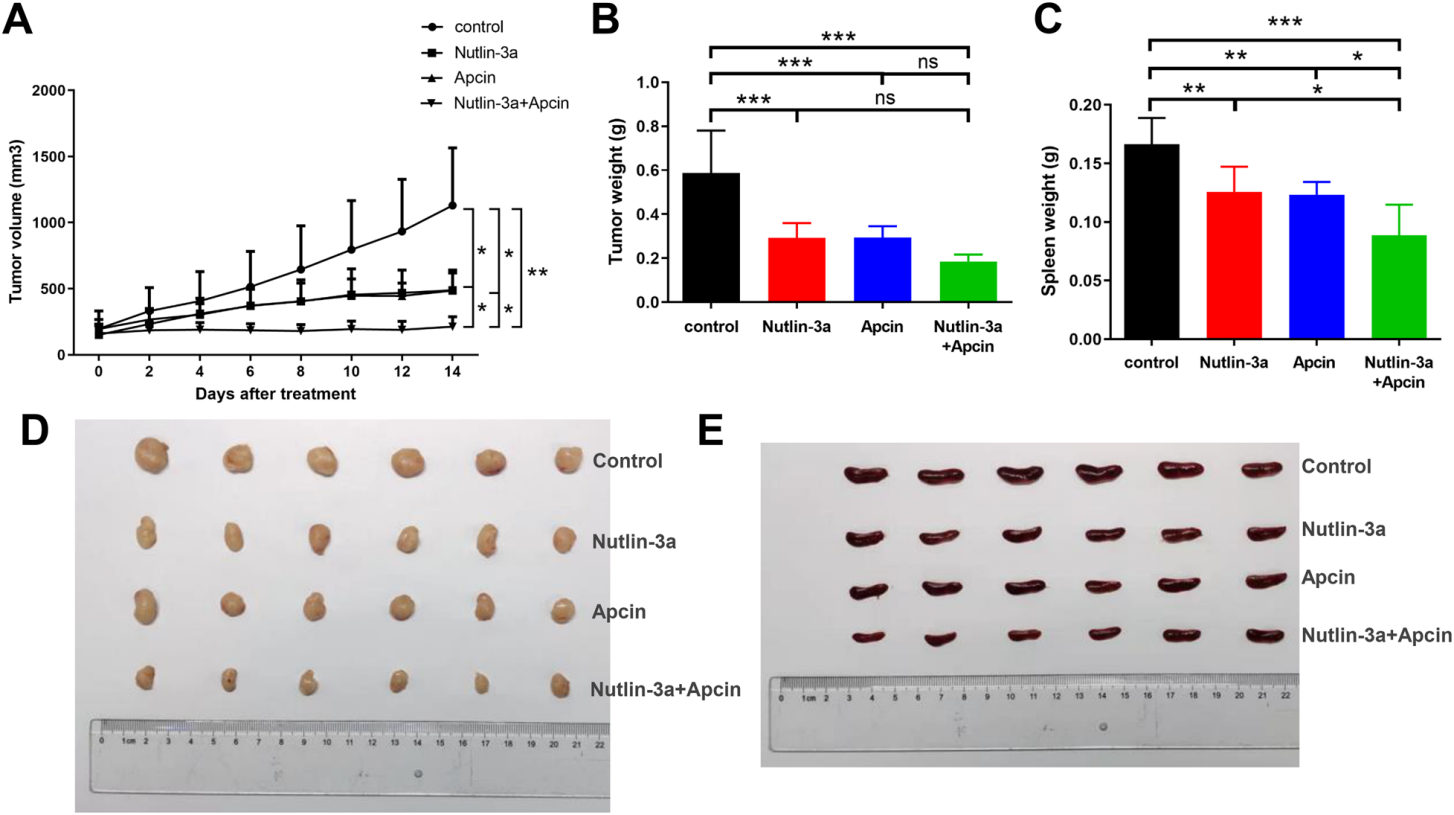
Treatment with nutlin-3a/apcin alone or in combination exerted anti-tumor effect in the Z138 cell-driven xenograft tumor model. **A** The tumor growth curves in the control group, the nutlin-3a group, the apcin group and nutlin-3a plus apcin group after treatment. Data were presented as mean ± SD. *P < 0.05, **P < 0.01. **B** Effect of treatment with nutlin-3a/apcin alone or in combination on tumor weight. Data were presented as mean ± SD. ***P < 0.001, ns meant* P* > 0.05. **C** Effect of administration with nutlin-3a/apcin alone or in combination on spleen weight. Data were presented as mean ± SD. *P < 0.05, **P < 0.01, ***P < 0.001. **D**, **E** Tumors (**D**) and spleens (**E**) in the four groups were harvested and photographed

We first evaluated the treatment efficacy in the four groups. Tumor growth in the nutlin-3a group, the apcin group and the combined group was significantly inhibited compared with the control group, and the combined group showed stronger tumor growth inhibition than respective single group (Fig. 9A and D). Besides, tumor weight in the three treatment groups was significantly lower than that in the control group (Fig. 9B). Surprisingly, we found treatment with nutlin-3a/apcin alone or in combination all reduced spleen weight, and combination treatment resulted in lower spleen weight than the two single treatment (Fig. 9C and E), indicating that combined injection could alleviate the splenomegaly caused by MCL modeling more effectively.

**Fig. 10:**
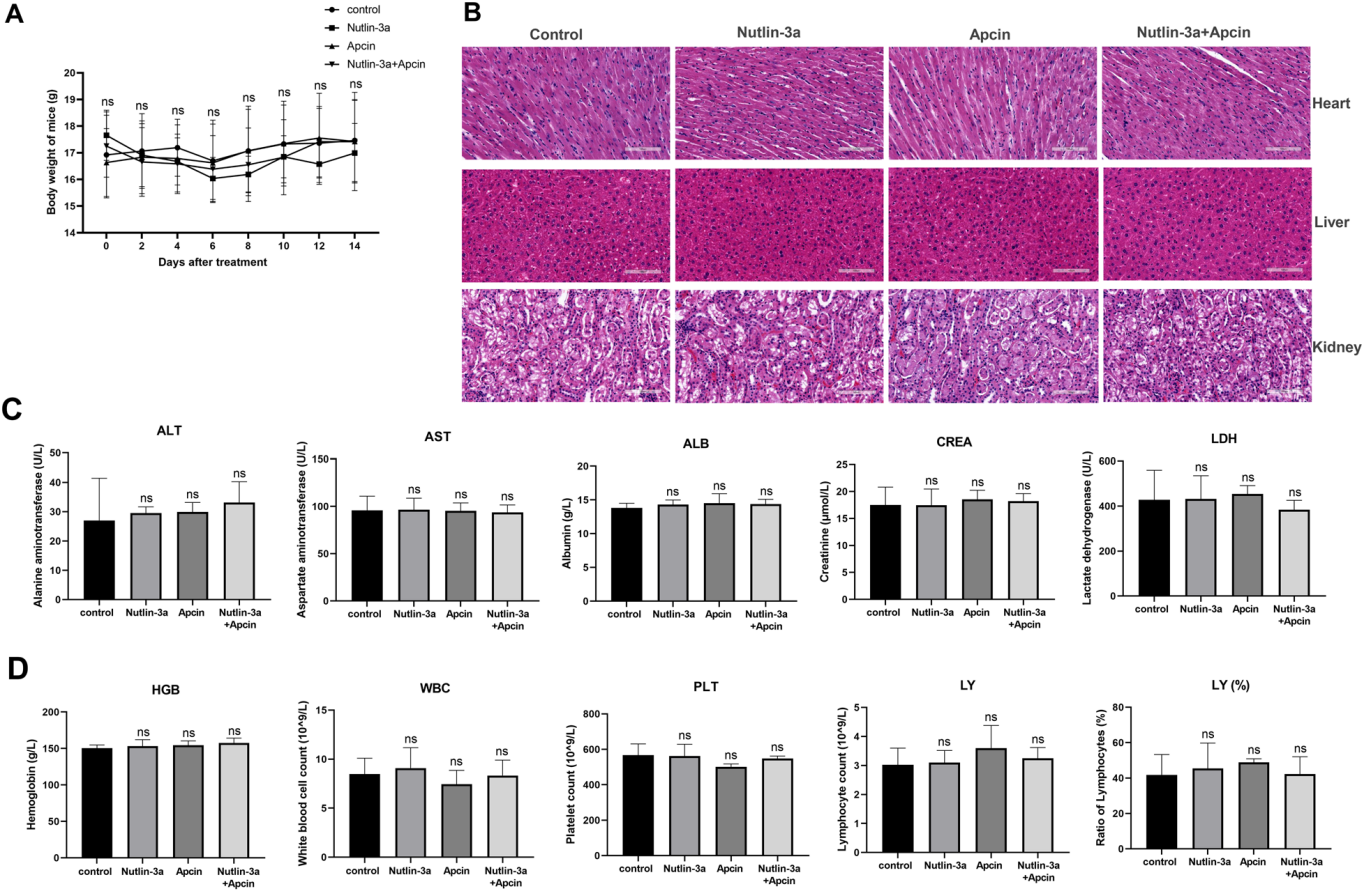
Injection of nutlin-3a/apcin alone or in combination was safe and tolerable in vivo. **A** The changes of mice body weight in the control group, the nutlin-3a group, the apcin group, and the nutlin-3a plus apcin group after treatment. ns meant* P* > 0.05. **B** Representative HE images of mice heart, liver, and kidney tissues in the four groups (× 200 magnification; scale bar, 100 μm). **C** Comparison of relevant indexes of the blood biochemistry test among the four groups. ns meant* P* > 0.05 compared with the control group. **D** Comparison of relevant indexes of the blood routine test among the four groups. ns meant* P* > 0.05 compared with the control group

In vivo safety of nutlin-3a and apcin administration was further discussed. Compared with the control group, nutlin-3a/apcin alone or in combination had no significant effect on body weight (Fig. 10A). HE staining showed that no obvious damage of heart, liver, and kidney was observed in the three treatment groups and the control group (Fig. 10B). The blood biochemistry results were also compared to determine whether there was abnormality in heart, liver and kidney function after treatment. Compared with the control group, serum alanine aminotransferase (ALT), aspartate aminotransferase (AST), albumin (ALB), creatinine (CREA) and lactate dehydrogenase (LDH) in the nutlin-3a group, the apcin group and the combined group showed no significant changes (Fig. 10C). Blood routine test was also performed on each mouse, and the results suggested that nutlin-3a/apcin alone or in combination had no significant effect on hemoglobin (HGB), white blood cell count (WBC), platelet count (PLT), lymphocyte count (LY) and ratio of lymphocytes (LY%) (Fig. 10D). These results illustrated the safety and tolerability of in vivo administration of nutlin-3a and apcin.

**Fig. 11:**
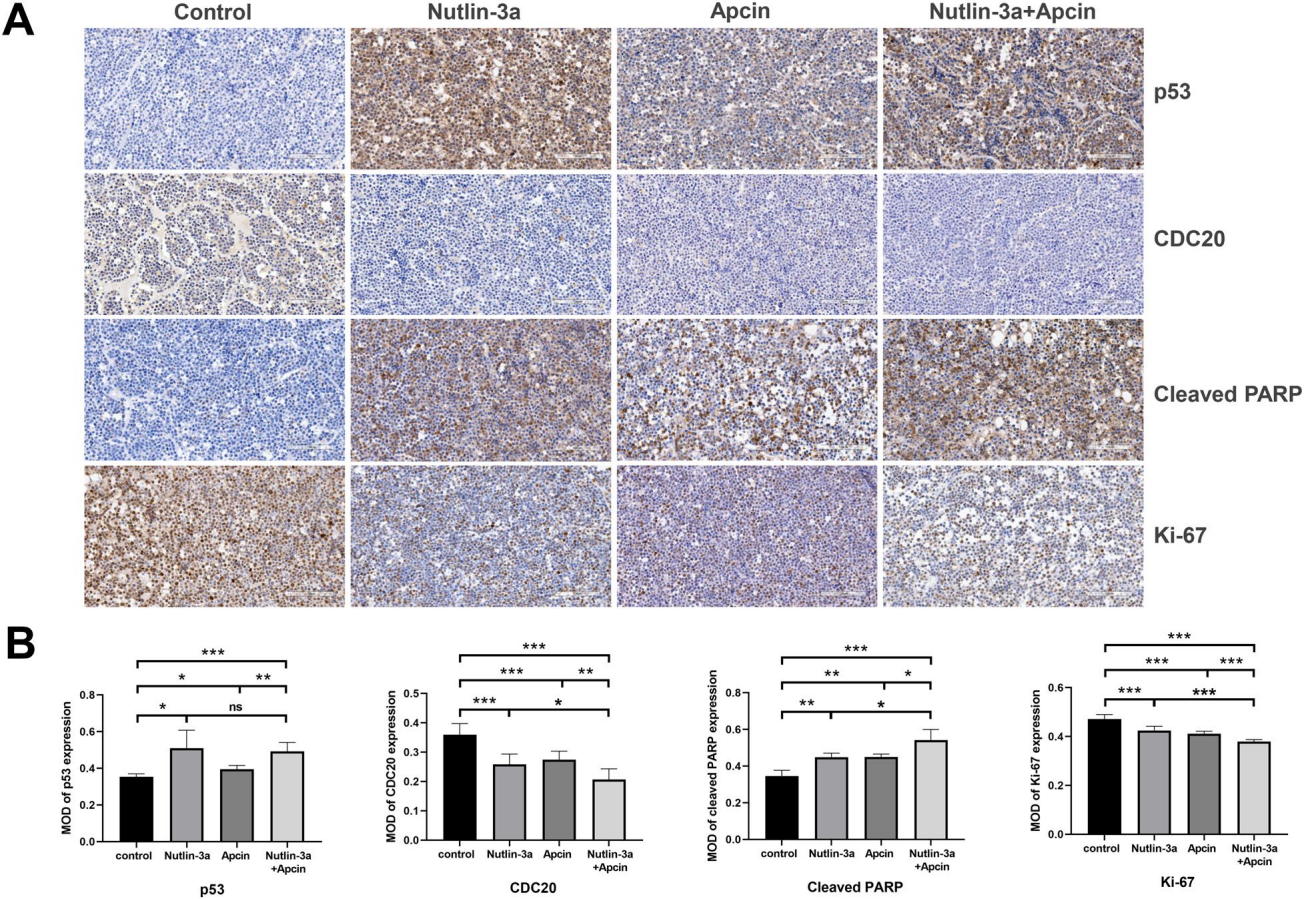
Targeted protein expression of the four mice groups. **A** Representative IHC images of p53, CDC20, cleaved PARP and K-i67 staining of tumor tissues in the control group, the nutlin-3a group, the apcin group, and the nutlin-3a plus apcin group (× 200 magnification; scale bar, 100 μm). **B** The protein expression of p53, CDC20, cleaved PARP and Ki-67 of tumor tissues were determined by IHC quantitative analysis in the control group, the nutlin-3a group, the apcin group, and the nutlin-3a plus apcin group, which were calculated by the MOD value. *P < 0.05, **P < 0.01, ***P < 0.001

The protein expression level of p53, CDC20, cleaved PARP and Ki-67 in the tumor tissues in these four groups was compared by IHC staining (Fig. 11A). As shown in Fig. 11B, p53 expression was significantly elevated after administration of nutlin-3a alone or combined with apcin, while p53 expression had no significant difference between the nutlin-3a group and the combined group. CDC20 expression was downregulated in the three treatment groups, and the expression level was lowest in the co-treatment group. The protein expression of cleaved PARP, an apoptosis index, was significantly increased in the three treatment groups, and the expression in the combined group was higher than that in the two single treatment group. On the contrary, tumor proliferation was significantly attenuated in the three treatment groups, accompanied by the decreased expression level of Ki-67, and the expression level was lowest in the combined group. Therefore, we validated combined administration of nutlin-3a and apcin promoted tumor apoptosis and suppressed tumor proliferation more potently than single administration.

## Discussion

Although more and more targeted drugs have been used for clinical trials in recent years to prolong the long-term survival of MCL patients and improve their clinical prognosis, most MCL patients still progress to relapsed/refractory cases [3, 38]. Therefore, studies on relevant markers in MCL pathogenesis may help to identify potential new targets for MCL therapy. Cell cycle disorder was the major event occurring in MCL, characterized by overexpression of cyclin D1, stimulation of cyclin-dependent kinase (CDK) 4 or 6 and depletion of CDK inhibitor p16 ^INK4^. These abnormalities ultimately disrupted the G1 phase in the cell cycle and promote the G1/S transition, leading to tumor proliferation in MCL [12, 39, 40]. Some clinical trials explored the potential of CDK inhibitors for MCL treatment. For example, the overall response rate (ORR) of Palbociclib, a selective CDK4/6 inhibitor, was 18% in MCL patients [41], while it increased to 21% and 67% after combination with bortezomib [42] and ibrutinib [43], respectively. Abemaciclib, another CDK4/6 inhibitor, obtained an ORR of 35.7% in relapsed/refractory MCL patients [44]. AT7519M, a pan-CDK inhibitor targeting CDK1/2/4/5/9, had an ORR of 27% in MCL patients [45]. Flavopiridol, another pan-CDK inhibitor targeting CDK1/2/4/6, got a partial remission rate of 11% in MCL patients [46]. Thus, the therapeutic efficacy of these clinical trials was not ideal, and patients usually suffered from adverse events such as neutropenia, thrombocytopenia and diarrhea.

There was an urgent need to identify other cell cycle-related therapeutic targets. Few studies explored the role of mitotic phase in MCL progression. CDC20, as a substrate-recruiting subunit, could bind to APC/C to form the activated APC/C-CDC20 complex, thereby causing metaphase-anaphase transition, mitotic exit and ubiquitination-mediated degradation of key substrates in cell cycle [15, 47, 48]. In addition to its pivotal role in regulating cell cycle progression, CDC20 has been reported to have oncogenic effect due to its overexpression in a broad spectrum of cancers. Meanwhile, CDC20 functioned as the prognostic factor in some tumors, and was closely related to the clinicopathological characteristics of tumor patients [16–27]. Based on the multiple roles of CDC20 in cell cycle and carcinogenesis, we considered its role in MCL pathogenesis. Maes et al. has ever demonstrated that APC/C was a new target for the treatment of MCL, while the effect of CDC20 on MCL cells was not explored in depth [34]. Guo et al. conducted a series of bioinformatics analysis on GSE93291 dataset and found that CDC20 was associated with the overall survival of MCL patients, but the authors did not further prove whether CDC20 was indeed an important target in MCL pathogenesis [49]. In the current study, we confirmed that CDC20 was upregulated at both mRNA and protein levels in MCL patients and MCL cell lines possessing either mutant p53 (Jeko and Mino) or wild type p53 (Z138 and JVM2). Among these cell lines, p53-mutant cells obtained higher CDC20 expression level than p53-wild type cells, which might be partly due to the loss of p53 regulation in p53-mutant cells. There was a positive correlation between cyclin D1 and CDC20 in the 51 patients with IHC staining, which meant the more serious the patient's condition, the higher CDC20 expression. We compared the clinical parameters between the high CDC20 expression group and the low CDC20 expression group in MCL patients with PBMCs and tumor sections, and revealed that higher expression of CDC20 implied poor treatment response, worse tumor staging, increased risk of bone marrow involvement and dismal prognosis in MCL patients. Since peripheral blood is easily available from clinical patients, it would be suitable to detect CDC20 expression in PBMCs in the future. Next, we explored the effect of CDC20 on the biological behaviors of MCL cells. The CDC20 inhibitor apcin, which blocked the CDC20-related substrates binding to CDC20, has been proved in several researches to have effect on tumor cell growth and drug sensitivity [50–53]. In this study, apcin treatment attenuated cell proliferation, migration and invasion, enhanced cell apoptosis, and induced G2/M arrest, explaining that CDC20 inhibition could exert anti-tumor activity in MCL.

We hoped to find the upstream regulators targeting CDC20 and discussed the specific mechanism of its influence on the development and progression of MCL by regulating CDC20. In addition to chromosomal translocation t (11; 14) (q13; q32), p53 inactivation was another common cytogenetic changes in MCL patients, accounting for 20% of newly diagnosed cases [40]. In our study, p53 mutation rate was 37.5% (9/24) in 24 MCL patients with peripheral blood samples. How to treat MCL patients with p53 mutation was challenging. One alternative mechanism of p53 inactivation was MDM2 overexpression, and MDM2 was the negative regulator of p53 [30, 54]. Therefore, activating p53 by inhibiting MDM2 might become a new method for MCL treatment. Notably, p53 was a recognized transcription factor, and targeted genes transcriptionally inhibited by p53 were usually upregulated in tumors and considered to be the targets of cancer therapy [31]. We found CDC20 was highly expressed in MCL, together with the fact that p53-mutant MCL cells had higher CDC20 expression and p53 inactivation was common in MCL, so we speculated that p53 might be the upstream regulator of CDC20. A previous study performed by Kidokoro et al. showed that p53 negatively regulated CDC20 expression through CDE–CHR elements in the CDC20 promoter. This regulation was in a p53-dependent manner, as CDC20 suppression by adriamycin-induced p53 elevation was observed only in p53 wild-type cells, not in p53 mutant and p53 null cells [31]. Banerjee et al. reported that under genotoxic stress, p53 transcriptionally downregulated CDC20 by directly binding to CDC20 promoter and promoted chromatin remodeling [55]. Another study using bioinformatics analysis concluded that in triple-negative breast cancer (TNBC), CDC20 was regulated by the p53 signaling pathway. After treatment with podophyllotoxin, p53 expression increased and CDC20 expression decreased in TNBC cell lines [32]. Sun et al. proved that CDC20 was a critical downstream factor of MDM2-p53 signaling pathway in diffuse large B-cell lymphoma, and knockdown of MDM2 resulted in upregulation of p53 and downregulation of CDC20 [27].

However, no research explained the interaction of p53 and CDC20 in MCL. We preliminarily discovered the negative correlation between p53 and CDC20 in GSE10793 and GSE93291, two GEO datasets of MCL patients. We also analyzed the relationship between p53 and CDC20 in our own cohort with 24 patients. Although the results had no statistical significance due to small sample size, a negative regulation trend was existed between p53 and CDC20. Nutlin-3a, an agent that inhibited MDM2 to reactivate p53, was chosen to further illustrate the relationship between p53 and CDC20 in MCL. We verified elevated expression of p53 and reduced expression of CDC20 in nutlin-3a-treated p53-wild type MCL cells, not p53-mutant MCL cells. This meant only p53 with activated function qualified to regulate CDC20. Moreover, nutlin-3a had inhibitory effect on MCL tumorigenesis similar as apcin in p53-wild type Z138 and JVM2 cells, indicating the important function of p53-CDC20 regulatory axis in MCL. In terms of mechanism, the dual-luciferase reporter gene assay and CUT&Tag technology validated that CDC20 was transcriptionally repressed by p53 via directly binding p53 to CDC20 promoter from upstream 492 bp to downstream 101 bp of TSS in MCL. These findings were consistent with previous studies, further suggesting the negative regulation of p53 on CDC20 might be universal in cancers.

As mentioned above, we uncovered the important role of p53 and CDC20 in MCL. Based on the clinical fact that cell cycle dysregulation and p53 inactivation were commonly occurred in MCL patients, we explored the combined effect of nutlin-3a and apcin in MCL. Theoretically, combined inhibition of nutlin-3a and apcin on CDC20 expression might have a stronger anti-MCL effect. In our study, combination of the two agents inhibited CDC20 protein expression to a great extent, and achieved better anti-proliferative and pro-apoptotic activities than single agent. Most importantly, no matter nutlin-3a or apcin were used alone or in combination, they had little effect on the cell viability of healthy PBMCs (Figs. 3A, 5A and 7A), demonstrating the tumor-specific inhibitory effect and safety for normal cells of the two agents in vitro. The therapeutic potential of nutlin-3a and apcin was also examined in vivo. Nutlin-3a/apcin alone or in combination prevented tumor growth and benefited the mice spleens by reversing the splenomegaly caused by MCL modeling, and the combination group showed the best efficacy. We confirmed that injection of nutlin-3a/apcin alone or in combination was safe and tolerable in vivo, as no significant abnormality was found in mice body weight, organ function, blood routine test and blood biochemistry test. IHC staining also revealed that CDC20 protein expression was lower in the combined treatment group than the single treatment group. Meanwhile, increased cleaved PARP expression and decreased Ki-67 expression were more apparent in the combined group than the single group. These data provided initial evidence for the clinical application of nutlin-3a and apcin.

## Conclusions

This study validated the essential role of CDC20 in MCL tumorigenesis as well as the mechanism of how p53 regulated CDC20 in MCL. We confirmed that CDC20 was upregulated in MCL, and high expression of CDC20 was related with poor clinicopathological features and prognosis of MCL patients. Both CDC20 inhibitor apcin and p53 agonist nutlin-3a could inhibit cell proliferation, migration and invasion, and induce apoptosis and cell cycle arrest in MCL cells. Combined treatment of nutlin-3a and apcin in vitro and in vivo enhanced anti-MCL activity. Therefore, dual-targeting p53 and CDC20 is promised to be a prospective MCL treatment strategy, which provides a new insight for MCL therapeutics.

## Data Availability

The datasets used and/or analysed during the current study are available from the corresponding authors on reasonable request.

## Abbreviations

MCL: Mantle cell lymphoma
CDC20: Cell division cycle 20 homologue
APC/C: Anaphase-promoting complex/cyclosome
PBMCs: Peripheral blood mononuclear cells
BMNCs: Bone marrow mononuclear cells
LRH: Lymph node reactive hyperplasia
CI: Combination index
MMP: Mitochondrial membrane potential
IHC: Immunohistochemiscal staining
HE: Hematoxylin–eosin staining
MOD: Mean optical density
MIPI: MCL International Prognostic Index
MIPI-c: Combined MCL International Prognostic Index
CR: Complete remission
PR: Partial remission
SD: Stable disease
PD: Progressive disease
OS: Overall survival
CDK: Cyclin-dependent kinase
